# Comparative Evaluation of Mechanical Properties Between CAD/CAM-Milled and 3D-Printed Dental Zirconia: A Systematic Review and Meta-Analysis

**DOI:** 10.3390/ma18225112

**Published:** 2025-11-11

**Authors:** Mohammed A. Alrabiah

**Affiliations:** Department of Prosthetic Dental Science, College of Dentistry, King Saud University, P.O. Box 60169-15, Riyadh 11545, Saudi Arabia; mohalrabiah@ksu.edu.sa

**Keywords:** 3D printing, bonding strength, CAD/CAM, fixed dental prostheses, flexural strength, Vickers hardness, zirconia

## Abstract

**Highlights:**

**What are the main findings?**

- Three-dimensionally printed zirconia achieves clinically acceptable strength, which justifies clinical use.
- Milled zirconia shows greater accuracy and reliability.
- Printing orientation influences flexural strength outcomes.
- Surface glazing reduces roughness in both materials.

**What are the implications of the main finding?**

- Three-dimensional printing can complement milling in dental restorations.
- Proper surface treatment enhances bonding performance.
- Additive methods suit complex shapes with less material waste.

**Abstract:**

The field of dental restorations continues to demand durable prosthetic materials with a focus on esthetic appeal. This systematic review and meta-analysis compared the mechanical properties and bonding performance of computer-aided design (CAD)/computer-aided manufacturing (CAM)-milled and three-dimensionally (3D) printed zirconia fixed dental prostheses. A systematic search of major databases identified 15 eligible recent in vitro studies. Random-effects meta-analyses (based on standard mean deviation) and heterogeneity (I^2^) and sensitivity analyses were performed. The meta-analysis showed no significant differences between the groups in flexural strength, hardness, density, bond strength, and fracture toughness. However, heterogeneity remained high, reflecting possible differences in the build orientation, additive manufacturing technique, and sintering protocols. A qualitative analysis of the literature also revealed that milled zirconia was generally associated with greater consistency in strength, hardness, and accuracy. Three-dimensionally printed zirconia, while more variable due to porosity and processing factors, frequently reached clinically acceptable values, with certain orientations achieving flexural and bonding strengths equal to or surpassing those of milled zirconia. Both fabrication methods benefited from surface treatments, and artificial aging confirmed stability within functional ranges. Overall, CAD/CAM-milled zirconia remains the benchmark for predictability; however, advances in additive manufacturing suggest a growing potential for 3D-printed zirconia in complex restorations.

## 1. Introduction

In recent decades, the dental restorative field has shifted significantly from using metal–ceramic systems to using multi-layered zirconia. Dental prosthetics, unlike other prosthetics, focus on the esthetic finish without compromising the mechanical requirements. Restorative systems have been largely dependent on ceramics, mainly due to the numerous advantages that they provide. Ceramic dental prosthetics are durable, chemically stable, and biocompatible and possess optical qualities comparable to those of real teeth [1,2]. However, a major disadvantage, which has been an inconvenience to patients, is the lack of appealing esthetic features. The telltale opacity or slight grayish tone can be difficult to ignore, which may explain why researchers have been so intent on finding alternatives that combine strength with appearance [1]. Zirconia, in many respects, serves as a suitable replacement for ceramic prosthetics. It offers mechanical toughness, while being compatible biologically, and is also stable chemically and offers optimum optical characteristics, much like dental ceramics [3,4]. That said, zirconia is not flawless; some clinicians note that it can appear slightly opaque compared to glass ceramics, which may be important in highly visible restorations. Lately, much of the attention has turned to yttria-stabilized zirconia (YSZ), particularly for crowns and fixed dental prostheses. Yttria content determines phase composition and properties: 3 mol% Y_2_O_3_ (3Y-TZP) is predominantly tetragonal and high-strength, 5 mol% (5Y-PSZ) is tetragonal–cubic (approximately 50:50) with greater translucency but lower strength, and 7 mol% approaches fully cubic with further translucency gains and strength trade-offs. YSZ has an impressive ability to withstand mechanical pressures due to its high strength (800–1200 MPa). The high strength helps in resisting any change in shape and size over time, which makes it suitable for posterior restorations, which must withstand higher occlusal loads [5]. There are numerous different types of dental zirconia, which leads to confusion about which type to choose for each restoration. Many different types of zirconia can be employed in the fabrication process of dental implants and fixtures. When mirror-polished, zirconia resists bacterial adhesion while having an increased adhesive affinity toward soft tissues. This is an added advantage in implant superstructures manufacturing, because it provides mechanical integrity while maintaining the esthetic component of dental implants [6].

One of the main approaches to producing zirconia restorations is the computer-aided design and computer-aided manufacturing (CAD/CAM) technology, especially through subtractive manufacturing (SM) technologies (milling). The term “subtractive” is used because the desired fixed dental prosthesis (FDPs) is fabricated by effectively “subtracting” the material from a prefabricated zirconia block [3,7]. One study has highlighted that CAD-CAM production systems offer many benefits in terms of high quality and precision for fixed dental restorations [8]. The error range is minimal, and the prevalence of microgaps is lower [9].

It is also worth noting that there have been rapid developments in three-dimensional (3D) printing for the healthcare sector, especially the dental sector. When designing dental restorations, it is important to keep in mind that there should be no compromise in the mechanical and physical properties, biocompatibility, and cost-effectiveness [10]. It is worth noting that 3D printing has carved out a place in dentistry, even extending to manufacturing diagnostic casts, temporary crowns, implant guides, and night guards. More recently, researchers have begun experimenting with the 3D printing of zirconia-based dental prosthetics. The actual printing methods cover an extensive range: stereolithography (SLA), selective laser sintering, fused deposition modeling, direct light processing (DLP), selective laser melting, and even direct inkjet printing [7].

Although many reviews have analyzed zirconia as a dental restoration material, very few have focused on the direct comparison between CAD/CAM-milled and 3D-printed zirconia. The issue also extends to the fact that many existing reviews overlook important parameters, including the recent advancements in additive manufacturing (AM) and bonding strength. The findings across many studies have also remained significantly inconsistent. Therefore, a systematic review and meta-analysis on this topic can clarify discrepancies (if any) and help determine whether 3D-printed zirconia can be a reliable alternative to CAD/CAM-milled zirconia.

## 2. Materials and Methods

### 2.1. Study Selection and Criteria

Articles were selected and the entire study was conducted in accordance with the Preferred Reporting Items for Systematic Reviews and Meta-Analyses (PRISMA) guidelines.

This systematic review was performed based on the following PICO question: In patients (or in vitro models) receiving zirconia-based FDPs, does 3D-printed dental zirconia demonstrate different mechanical properties and bonding strength compared to CAD/CAM-milled zirconia?

More specifically, the PICO stands for the following:

**Population (P)** 

- Patients receiving fixed dental prostheses (crowns, FDPs, and implant-supported restorations) fabricated using zirconia.
- Extracted human teeth, standardized dental models, or laboratory-prepared specimens of zirconia, keeping in mind that relevant bonding or mechanical data that complement clinical findings are available.

**Intervention (I)** 

- Different 3D printing techniques (e.g., SLA, DLP, and binder jetting) with zirconia-based materials.

**Comparison (C)** 

- CAD/CAM milling involving fabrication from pre-sintered zirconia blocks using SM.

**Outcomes (O)** 
***Mechanical Outcomes*** 

○ Flexural strength.
○ Fracture toughness.
○ Hardness.
○ Density.

***Bonding Strength*** 

○ Shear bond strength.
○ Adhesion to resin cements or veneering ceramics.

### 2.2. Search Strategy

A systematic search was carried out across multiple databases, including PubMed, Embase, Web of Science, Scopus, Cochrane Library, and Google Scholar (to capture gray literature). In addition, a manual review of the supplementary sources was undertaken.

The search strategy was implemented by combining a wide range of terms such as “zirconia,” “yttria-stabilized zirconia,” “zirconium oxide,” “Y-TZP,” “3Y-TZP,” “5Y-PSZ,” “CAD/CAM,” “computer-aided design,” “computer-aided manufacturing,” “milling,” “subtractive manufacturing,” “3D printing,” “additive manufacturing,” “rapid prototyping,” “stereolithography,” “digital light processing,” “selective laser sintering,” “mechanical properties,” “flexural strength,” “fracture toughness,” “hardness,” “Vickers hardness,” “bonding strength,” “adhesion,” “shear bond,” and “tensile bond.”

Commercial product names and clinical terminology relevant to ceramic processing techniques and performance outcomes were also incorporated to broaden coverage and minimize omissions.

### 2.3. Inclusion and Exclusion Criteria

#### 2.3.1. Inclusion Criteria

Original research studies, conducted as in vitro lab studies, comparing CAD/CAM-milled and 3D-printed/AM dental zirconia, were included. The present review included all relevant reports that highlighted mechanical properties, including flexural strength, fracture toughness, Vickers/Knoop hardness, elastic modulus, and Weibull modulus, or even bonding/adhesion outcomes highlighting shear/bond strength to resin cements, pull-off, and microtensile strength.

Research studies that focused on zirconia-based ceramics, especially yttria-stabilized zirconia (3Y/4Y/5Y), were chosen. The language was restricted to English and English translations. No filter was used for the publication data range. Studies that reported sufficient data with numerical results (mean ± SD or median/IQR) or clear qualitative comparisons were included. For meta-analysis candidates, the reporting of mean ± SD values and sample size was required for inclusion.

#### 2.3.2. Exclusion Criteria

Research studies that do not focus on comparing milled and 3D-printed zirconia (e.g., only one technique without a comparator) were explicitly excluded. Reports on different materials (e.g., lithium disilicate and alumina) without zirconia data, case reports, editorials, conference abstracts without usable data, and patents were not included. Similarly, studies using non-dental zirconia compositions, where results are not applicable to dental restorations, and studies that do not report the mechanical/bonding outcomes of interest were excluded.

### 2.4. Risk-of-Bias Assessment

Risk-of-bias assessment was carried out on the selected articles to determine the study quality. It was assessed using the Risk of Bias tool for Pre-Clinical Dental Material Research (RoBDEMAT) tool [11] and the modified Consolidated Standards of Reporting Trials (CONSORT) scale for in vitro dental materials [12].

The RoBDEMAT tool has four different domains with respect to the following types of bias:

D1—Bias in planning and allocation.

D2—Bias in sample/specimen preparation.

D3—Bias in outcome assessment.

D4—Bias in data treatment and outcome reporting.

The four domains have a total of nine items, including control group, sample randomization, standardization, and statistical analysis. Articles were graded against each of the nine key items as follows:

- A—Adequately performed/reported.
- I—Insufficiently performed/reported.
- N—Not performed/reported.
- NA—Not applicable.

The review also employed the modified CONSORT scale to assess each article against 14 different criteria. These included a structured summary of trial design, methods, results, and conclusions; scientific background and rationale; specific objectives; intervention; outcomes; sample size determination; randomization sequence generation; allocation; implementation; blinding; statistical methods; outcomes; limitations; funding; and trial protocol registration. The studies were rated based on the number of criteria met.

### 2.5. Meta-Analysis and Publication Bias Assessment

Meta-analyses were performed using JASP software (version 0.95.1; JASP Team, Amsterdam, The Netherlands). The random-effect model was used to perform the meta-analyses, and a forest plot was generated for each of the analyses. Moderator and sensitivity analyses were performed to understand the sources of heterogeneity in cases of high heterogeneity among the studies. Additionally, a subgroup analysis was performed, where deemed necessary. For assessing publication bias, a funnel plot was generated and visually analyzed, Egger’s test was performed, and Duval and Tweedie trim-and-fill analysis was conducted.

## 3. Results

### 3.1. Study Selection

**Figure 1:**
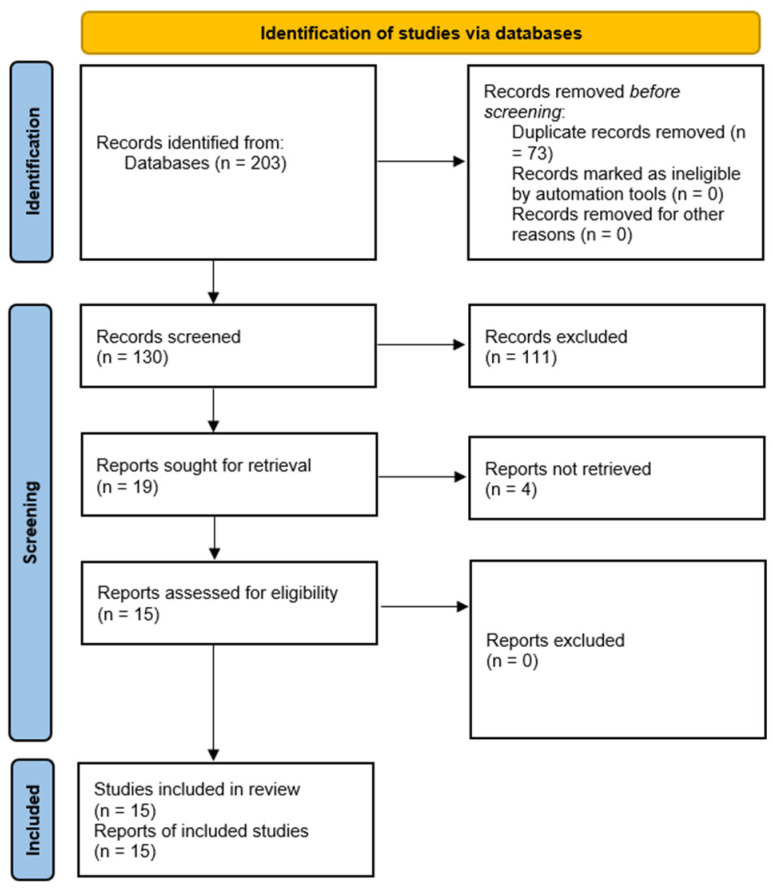
A PRISMA flow diagram schematically explaining the study screening and identification process.

A total of 203 studies were identified initially through database searching. Among them, 73 were duplicate entries; thus, 130 exclusive entries remained. A total of 111 studies were excluded from the analyses based on the specified inclusion and exclusion criteria, and 19 studies remained for assessment. Overall, four studies had to be excluded because the full text was not accessible. Finally, 15 studies (all in vitro studies) were included for the systematic review and meta-analysis. Figure 1 shows the screening and identification process schematically (PRISMA flow diagram). Furthermore, the values of the various parameters reported in these articles are presented in Table 1.

### 3.2. Study Quality Assessment

The findings of the risk-of-bias assessment—conducted using both RoBDEMAT and modified CONSORT tools—are presented in Table 2 and Table 3, respectively. Only one study randomized the samples or reported this aspect of the research [25]. Although randomization is less common in in vitro studies involving dental materials, it is recommended because it limits selection bias and balances mild material variability. Further, only one study blinded the test operator to group allocation or explicitly reported blinding [19]. In addition, three articles did not discuss the rationale behind choosing the sample size explicitly [17,22,23]. All articles provided enough rationale for the studies and explicitly stated the aims of the research. All articles also reported the limitations of their respective studies and explicitly provided information regarding funding. However, some studies did not provide information regarding the print orientation [16,17,23,24]. Additionally, some studies did not report on the sintering cycle or aging. Notably, sintering protocols for 3D-printed zirconia are longer because of the additional debonding step. Some also failed to report the surface condition [16,19]. In sum, all articles had a moderate level of quality and bias.

### 3.3. Qualitative Analysis

A total of 15 in vitro studies published between 2022 and 2025 were reviewed. In Korea, studies were performed by Cho et al., Kim et al., and Moon et al. [18,22,23]. In Europe, research was conducted by Alhotan et al., Bömicke et al., Hetzler et al., Refaie et al., and Zenthöfer et al. [15,17,21,24,25,26]. In the USA, work was carried out by Bergler et al. and Giugliano et al. [16,19]. In Saudi Arabia, studies were published by Abualsaud et al., Hajjaj et al., Alageel et al., and Hassan et al. [7,13,14,20].

#### 3.3.1. Mechanical Properties

Among the studies reviewed, 3D-printed zirconia consistently showed mechanical properties that were within clinically acceptable limits, often similar to milled zirconia in terms of flexural strength and fracture resistance [14,16,18,25]. However, printed specimens showed a greater variation in results, mainly due to porosity, microcracks, and print layer defects observed under a scanning electron microscope (SEM) [13,20]. Furthermore, color infiltration and two-step sintering slightly lowered the flexural strength of printed specimens [25]. Additionally, surface polishing did not significantly change the flexural strength of printed specimens [25]. It is also important to note that printing orientation had a significant effect on the mechanical parameters: for example, specimens printed horizontally reached flexural strength values equal to or in some cases higher than milled zirconia [21], whereas vertically printed specimens were weaker, although they still met the ISO 6872 standard [13]. In terms of toughness and hardness, milled zirconia consistently performed better than printed specimens [20,24]. Thermocycling and mechanical fatigue (artificial aging techniques) reduced the fracture toughness and hardness in both groups, and in printed zirconia, a significant decrease was observed [15]. Nevertheless, both materials retained values within the acceptable clinical ranges following aging [15,16].

#### 3.3.2. Fracture Toughness and Hardness

The fracture toughness and hardness of milled zirconia were consistently better than those of 3D-printed zirconia [20,24]. Thermocycling and mechanical fatigue induced artificial aging, which further decreased the fracture toughness and hardness of both groups; that said, the decrease was more pronounced in printed zirconia restorations [15]. However, the values of both materials remained within the acceptable clinical range after the aging protocols [15,16]. For real restorations, printed and milled restorations had comparable fracture resistance, especially on stiff support (Co-Cr abutments) or at a wall thickness of 0.6–0.8 mm; a wall thickness of 0.4 mm should be used cautiously because first-crack cusp forces can fall in the 140–200 N range [26].

#### 3.3.3. Surface Properties

Compared to milled zirconia, 3D-printed zirconia had higher surface roughness and marginally lower hardness [13,20]. Again, the printing orientation had a considerable effect on surface properties as well, with the highest surface roughness observed in tilted and vertically printed specimens [13]. In both groups, surface glazing proved to be effective in reducing roughness [20]. However, it should be noted that while surface glazing can transiently reduce roughness, accurate multistep polishing typically yields more durable smoothness and is preferred for long-term roughness control. In this study, surface finishing protocols considered glazing and multi-step polishing; thus, immediate roughness reduction (glaze) should be distinguished from more durable roughness control (polishing) when interpreting bond and wear implications. SEM was used to observe the structural flaws of printed zirconia, including voids and interlayer bandages, whereas milled zirconia had a more compact and uniform microstructure [14,16].

#### 3.3.4. Accuracy and Dimensional Fit

Milled zirconia showed greater accuracy than printed zirconia, with better internal and marginal adaptation and lower root mean square values (approximately 15–25 μm for milled zirconia and 40–50 μm for printed zirconia) [19,23]. However, restorations produced by AM were still within the clinically acceptable limits [13,23]. The slight inaccuracies observed in the printed specimens were mainly due to factors such as support removal, slurry shrinkage, and the inherent resolution limits of the printer.

#### 3.3.5. Bonding and Adhesion

Bond strength testing showed that 3D-printed zirconia could achieve resin cement adhesion comparable to milled zirconia, especially when treated with tribochemical silica coating (TSC) [7,17]. Although aging reduced the bond strength in both groups, most values remained above the 10 MPa threshold considered clinically acceptable. For veneering applications, AM showed a greater porcelain bond strength than SM. SEM confirmed these findings: stronger porcelain–zirconia interfaces in the printed specimens [23].

#### 3.3.6. Aging, Long-Term Durability, and Esthetics

Artificial aging by thermocycling and chewing simulation showed that both printed and milled zirconia maintained clinically acceptable properties after aging [16,18]. However, printed zirconia was more susceptible to degradation, particularly in terms of fracture toughness and bonding reliability [15]. Differences related to printing orientation persisted even after aging; horizontally printed specimens maintained greater stability than vertically printed specimens [13,21], The esthetic aspects remain poorly studied. One study found that milled zirconia had significantly higher translucency than printed zirconia and may be more appropriate for posterior restorations, where strength is prioritized over appearance.

### 3.4. Meta-Analysis

#### 3.4.1. Vickers Microhardness

**Figure 2:**
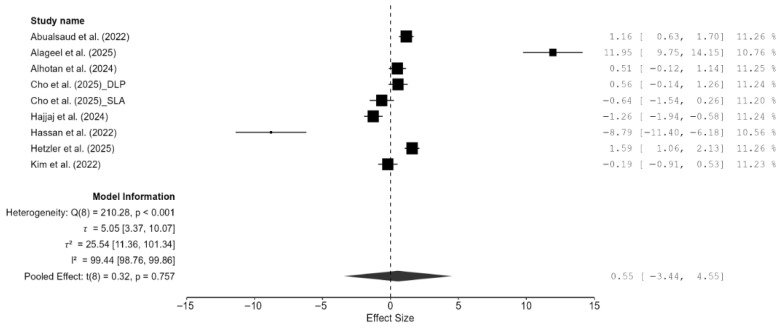
Forest plot of Vickers hardness analyzed by the random-effect model. The black squares represent the differences in Vickers hardness between the 3D-printed groups and the CAD/CAM groups in individual studies, and the area of each square indicates the sample size (study weight). The solid line depicts a 95% confidence interval of the difference. The diamond at the bottom of the graph shows the pooled estimate and uncertainty. The vertical line indicates the line of no effect, meaning there is no difference between the 3D-printed groups and the CAD/CAM groups [7,13,14,15,18,20,21,22].

**Figure A1:**
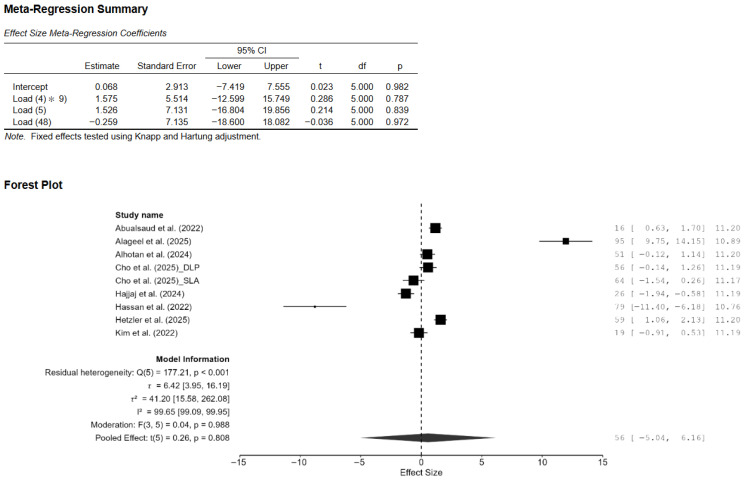
Effect of applied load on the difference in the mean effect size of Vickers hardness. CI: confidence interval, DLP: direct light processing, SLA: stereolithography [7,13,14,15,18,20,21,22].

**Figure A2:**
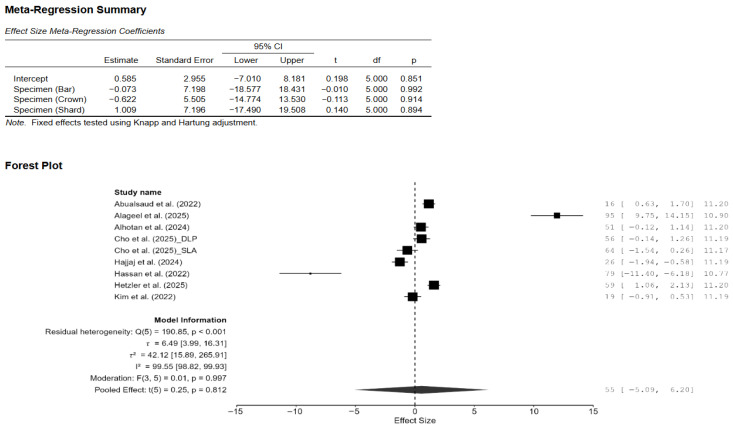
Effect of specimen shape on the difference in the mean effect size of Vickers hardness. CI: confidence interval, DLP: direct light processing, SLA: stereolithography [7,13,14,15,18,20,21,22].

**Figure A3:**
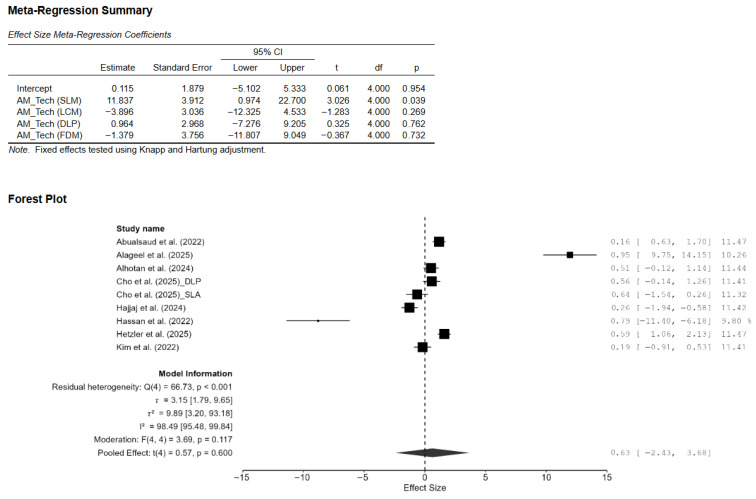
Effect of AM technology on the difference in the mean effect size of Vickers hardness. CI: confidence interval, DLP: direct light processing, SLA: stereolithography, AM: additive manufacturing, SLM: selective laser melting, LCM: lithography-based ceramic manufacturing, FDM: fused deposition modeling [7,13,14,15,18,20,21,22].

**Figure A4:**
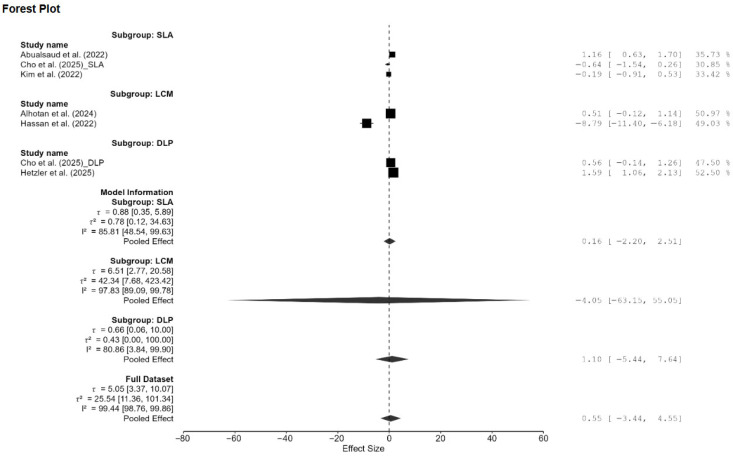
Subgroup analysis of Vickers hardness based on AM technology. DLP: direct light processing, SLA: stereolithography, LCM: lithography-based ceramic manufacturing; AM: additive manufacturing [13,15,18,20,21,22].

**Figure A5:**
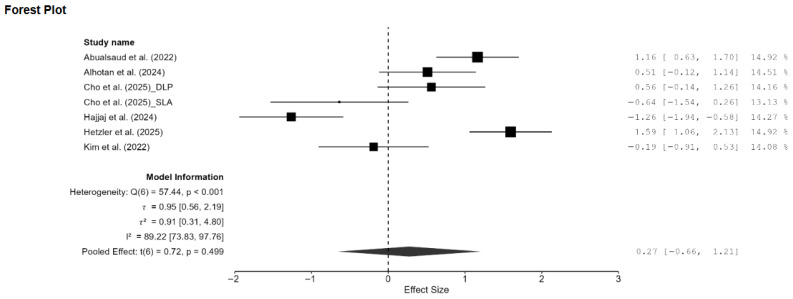
Forest plot of the sensitivity analysis on Vickers hardness performed by removing two outlier studies [14,20]. DLP: direct light processing, SLA: stereolithography [13,15,18,20,21,22].

A total of eight studies, out of the 15 studies included in the meta-analysis, reported Vickers hardness [7,13,14,15,18,20,21,22]. The pooled standardized mean difference (SMD) was found to be small and not statistically significant (SMD = 0.55; 95% CI: [−3.44, 4.55]; *p* = 0.76; Figure 2). Additionally, a very high heterogeneity was noted among the studies (I^2^ = 99.44%; 95% CI: 98.76–99.86%; *p* < 0.001). Thus, the author decided to perform a moderation analysis to understand the factors influencing the heterogeneity. Three moderators were used: AM technology, specimen type, and applied load. However, none of the prespecified moderators had a meaningful influence on the heterogeneity (Figure A1, Figure A2 and Figure A3). Additionally, subgroup meta-analyses based on AM technology were all non-significant, and there was no reduction in the heterogeneity or change in the inference (Figure A4). Furthermore, leave-one-out [14] and leave-two-out sensitivity analyses [14,20] conducted based on Cook’s distance (>2.0) revealed no change in the inference or heterogeneity (Figure A5). These results suggest that the heterogeneity stems from factors beyond the prespecified ones. Unfortunately, the exact factors causing the heterogeneity could not be determined because information regarding some of the other possible factors (e.g., build orientation, paste systems used in the AM procedure, yttria content, grain size, porosity, and sintering/aging protocols) was not available for all studies included in the meta-analysis. Crown-based SLA/DLP studies with 1 kgf/15 s loads reported hardness spanning approximately from 1442 HV to 1533 HV compared to approximately 1474 HV for milled crowns [18], while a DLP 5Y study showed HV0.5 of approximately 1590 [21], and an SLA–SM 3Y comparison found approximately 11.5–11.6 GPa with no significant difference [22], creating opposing effects that inflate the pooled variance.

**Figure 3:**
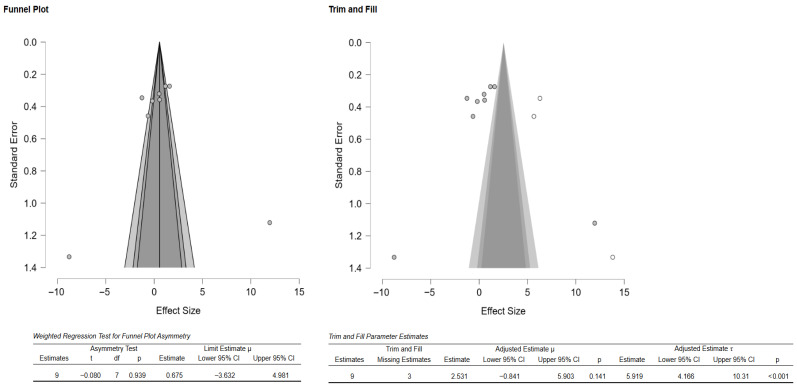
Funnel plot depicting the standard error by mean of Vickers hardness. The black dots in the trim-and-fill analysis indicate the studies identified in the systematic review, and the white dots represent the new studies that were imputed using the Duval and Tweedie trim-and-fill method. CI: confidence interval.

Additionally, the author performed publication bias analyses (Figure 3). The funnel plot showed that there was a potential publication bias or small-study effects. However, the weighted regression test (Egger’s test) did not confirm significant asymmetry (Figure 3). Additionally, the Duval and Tweedie trim-and-fill analysis suggested up to three missing studies, and the adjustment did not significantly change the overall effect, as indicated by the still wide confidence intervals and overlapping zero (Figure 3). There was high heterogeneity as well, meaning effect sizes varied considerably across studies. Thus, the evidence of possible bias exists, but results remain inconclusive and highly heterogeneous.

#### 3.4.2. Flexural Strength

**Figure 4:**
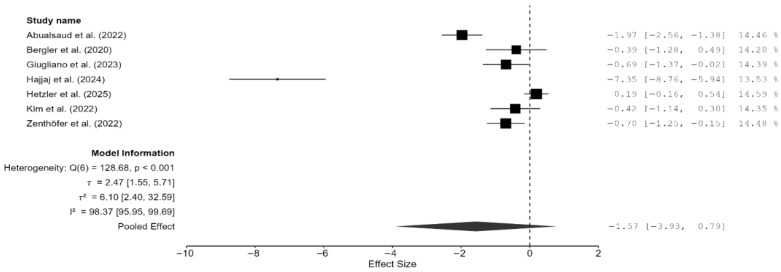
Forest plot of flexural strength analyzed by the random-effect model. The black squares represent the difference in flexural strength between the 3D-printed groups and the CAD/CAM groups in individual studies, and the area of each square indicates the sample size (study weight). The solid line depicts a 95% confidence interval of the difference. The diamond at the bottom of the graph shows the pooled estimate and uncertainty. The vertical line indicates the line of no effect, meaning there is no difference between the 3D-printed groups and the CAD/CAM groups [7,13,16,19,21,22,25].

**Figure 5:**
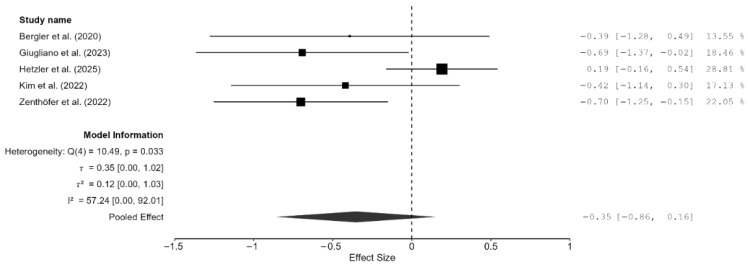
Forest plot of the sensitivity analysis performed by removing two outlier studies (Hajjaj et al., (2024) [7] and Abualsaud et al., (2022) [13]). The black squares represent the difference in flexural strength between the 3D-printed groups and the CAD/CAM groups in individual studies, and the area of each square indicates the sample size (study weight). The solid line depicts a 95% confidence interval of the difference. The diamond at the bottom of the graph shows the pooled estimate and uncertainty. The vertical line indicates that there is no difference between the 3D-printed groups and the CAD/CAM groups [16,19,21,22,25].

Of the 15 studies, a total of seven studies reported flexural strength [7,13,16,19,21,22,25]. The pooled effect size was negative but not statistically significant (SMD = −1.57 GPa; 95% CI: [–3.93, 0.79]; *p* = 0.16; Figure 4). However, it should be noted that between-study heterogeneity was very high (I^2^ = 98.37%; 95% CI: 95.95–99.69%; *p* < 0.001). Thus, a moderation analysis was performed for this parameter as well. However, none of the included moderators (testing method, specimen type, and AM technique) had any meaningful impact on heterogeneity. That said, when two studies [7,13], which provided highly varying values, were excluded from the meta-analysis, the heterogeneity reduced substantially (I^2^ = 57.25%). However, the pooled effect remained non-significant (SMD = –0.35 GPa; 95% CI: [–0.86, 0.16]; *p* = 0.13; Figure 5). Thus, it is evident from these findings of the sensitivity analysis that while these two studies contributed disproportionately to the heterogeneity, they did not impact the underlying effect; in fact, the effect size was much closer to zero.

#### 3.4.3. Density

**Figure 6:**
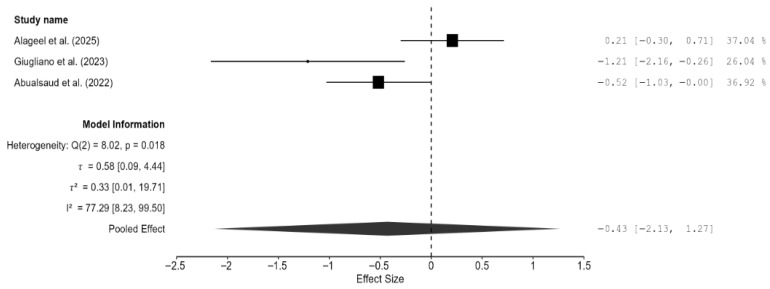
Forest plot of density analyzed by the random-effect model. The black squares represent the differences in density between the 3D-printed groups and the CAD/CAM groups in individual studies, and the area of each square indicates the sample size (study weight). The solid line depicts a 95% confidence interval of the difference. The diamond at the bottom of the graph shows the pooled estimate and uncertainty. The vertical line indicates the line of no effect, meaning there is no difference between the 3D-printed groups and the CAD/CAM groups [13,14,19].

**Figure A6:**
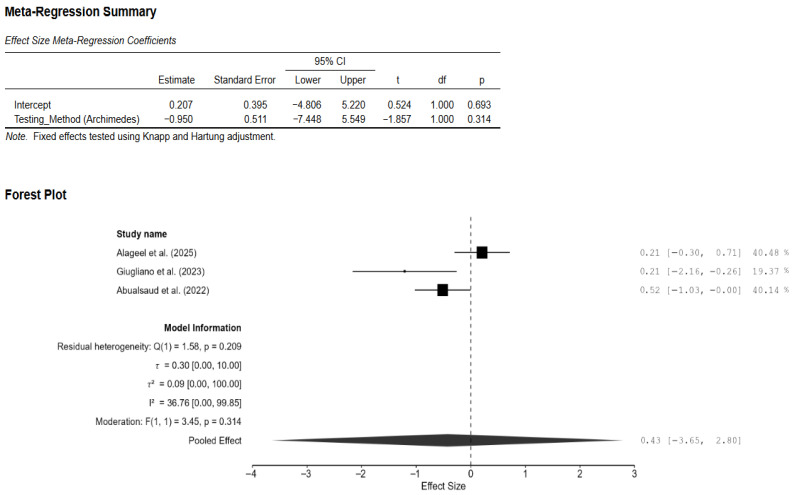
Effect of density testing method on the difference in the mean effect size of density. CI: confidence interval [13,14,19].

Three studies were included for analyzing the difference in density between the CAD/CAM and 3D-printed groups [13,14,19]. The pooled effect on density was very small but non-significant (−0.43; 95% CI: [−2.13, 1.27]; *p* = 0.39; Figure 6). There was significant heterogeneity among the three studies (I^2^ = 77.29%; 95% CI: [8.23–99.51%]; *p* = 0.02). Publication bias analyses revealed some bias in the studies. However, it should be noted that with only three studies, Egger’s and trim-and-fill analyses could misinterpret genuine heterogeneity as bias. Interestingly, the testing method used (water displacement vs. gravimetric analysis) significantly reduced the heterogeneity to 36.76% (Figure A6).

#### 3.4.4. Bond Strength

**Figure 7:**
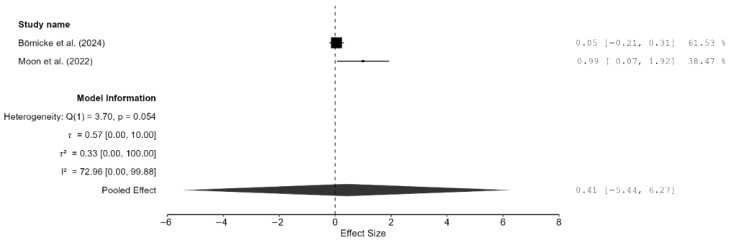
Forest plot of bond strength analyzed by the random-effect model. The black squares represent the differences in bond strength between the 3D-printed groups and the CAD/CAM groups in individual studies, and the area of each square indicates the sample size (study weight). The solid line depicts a 95% confidence interval of the difference. The diamond at the bottom of the graph shows the pooled estimate and uncertainty. The vertical line indicates the line of no effect, meaning there is no difference between the 3D-printed groups and the CAD/CAM groups [17,23].

Only two studies reported bond strength [17,23]. The pooled effect on bond strength was small but non-significant, and unsurprisingly, the confidence interval was very wide (SMD = 0.41 MPa; 95% CI: [−5.44, 6.27; *p* = 0.54; Figure 7). Furthermore, there was moderate heterogeneity (I^2^ = 72.96%); however, the CI was very wide, and this result is meaningless because there were only two studies. Additionally, publication bias analyses were not performed because of the extremely small number of studies included.

A review of both studies individually revealed that both interventions (CAD/CAM and 3D-printing) resulted in clinically acceptable bond strengths with resin cement. Additionally, the pooled mean values remained above the clinical threshold of 10 MPa, even after aging. Furthermore, surface treatment (TSC) and proper cement choice were found to be more influential than the fabrication method itself. These two studies suggest that printed zirconia is not inferior to milled zirconia when properly conditioned. Moon et al. suggested that 3D-printed zirconia restorations may provide even better porcelain bond strength, albeit at the cost of manufacturing accuracy [23]. Thus, printed zirconia appears to be a viable alternative but requires more refined protocols for fit and finishing.

#### 3.4.5. Fracture Toughness and Resistance

**Figure 8:**
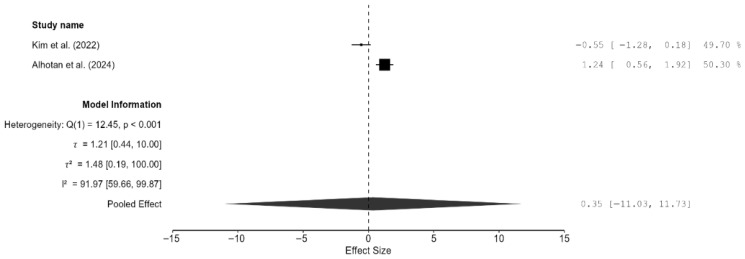
Forest plot of fracture toughness analyzed by the random-effect model. The black squares represent the difference in fracture toughness between the 3D-printed groups and the CAD/CAM groups in individual studies, and the area of each square indicates the sample size (study weight). The solid line depicts a 95% confidence interval of the difference. The diamond at the bottom of the graph shows the pooled estimate and uncertainty. The vertical line indicates that there is no difference between the 3D-printed groups and the CAD/CAM groups [15,22].

Both studies reported fracture toughness clustered near the line of null effect, suggesting comparable effects rather than superiority of one method over the other [15,22]. The pooled effect was very small, but the result was not significant and the confidence intervals were very wide (SMD = 0.35; 95% CI: [−11.03, 11.73]; *p* = 0.76; Figure 8). An individual review of the two studies revealed that 3D-printed zirconia specimens matched or exceeded milled zirconia specimens in terms of fracture toughness. In the study by Kim et al., there was no significant difference in fracture toughness (*p* = 0.1) between the two techniques, and both groups had a flexural strength greater than 800 MPa, which meets the ISO 6872 Class 5 requirements [22]. Alhotan et al. showed that 3D-printed specimens had a higher fracture toughness (6.07 MPa·m½) than milled ones (4.46 MPa·m½), and this result was maintained even after aging, although the magnitude of the difference reduced slightly [15].

**Figure 9:**
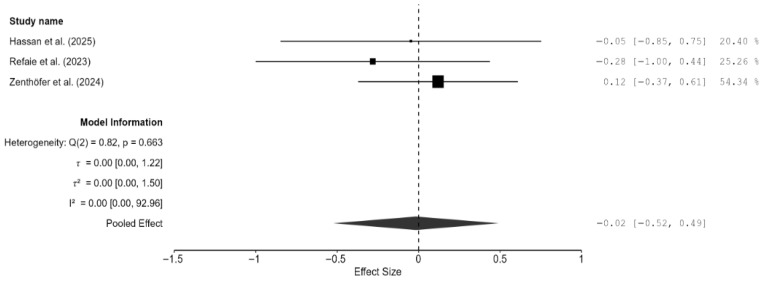
Forest plot of fracture resistance analyzed by the random-effect model. The black squares represent the differences in fracture resistance between the 3D-printed groups and the CAD/CAM groups in individual studies, and the area of each square indicates the sample size (study weight). The solid line depicts a 95% confidence interval of the difference. The diamond at the bottom of the graph shows the pooled estimate and uncertainty. The vertical line indicates the line of no effect, meaning there is no difference between the 3D-printed groups and the CAD/CAM groups [20,24,26].

Similar to fracture toughness, fracture resistance was reported by two studies only [20,24,25]. Overall, milled zirconia restorations had a marginally higher fracture resistance than 3D-printed restorations; however, the result was not significant (SMD = −0.02; 95% CI: [−0.52, 0.49]; *p* = 0.91; Figure 9). There was no heterogeneity between the two studies. Weibull analysis also showed higher modulus values for milled zirconia restorations, which translates to a more predictable fracture performance, whereas 3D-printed zirconia had a greater variability but a similar mean strength.

#### 3.4.6. Meta-Analysis of the Composite Mechanical Outcome

**Figure 10:**
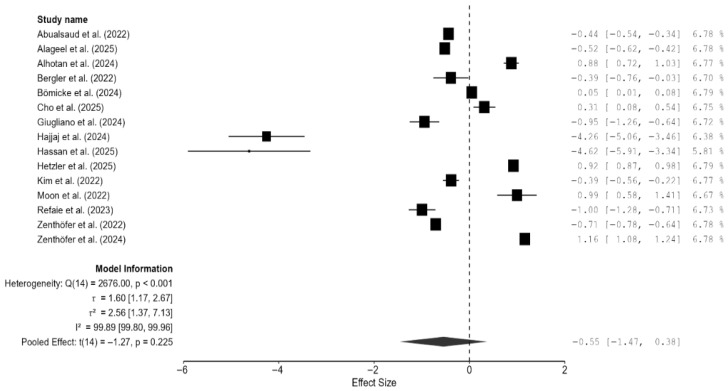
Forest plot of the composite mechanical outcome analyzed by the random-effect model. The black squares represent the differences in the composite outcome between the 3D-printed groups and the CAD/CAM groups in individual studies, and the area of each square indicates the sample size (study weight). The solid line depicts a 95% confidence interval of the difference. The diamond at the bottom of the graph shows the pooled estimate and uncertainty. The vertical line indicates the line of no effect, meaning there is no difference between the 3D-printed groups and the CAD/CAM groups [7,13,14,15,16,17,18,19,20,21,22,23,24,25,26].

**Figure 11:**
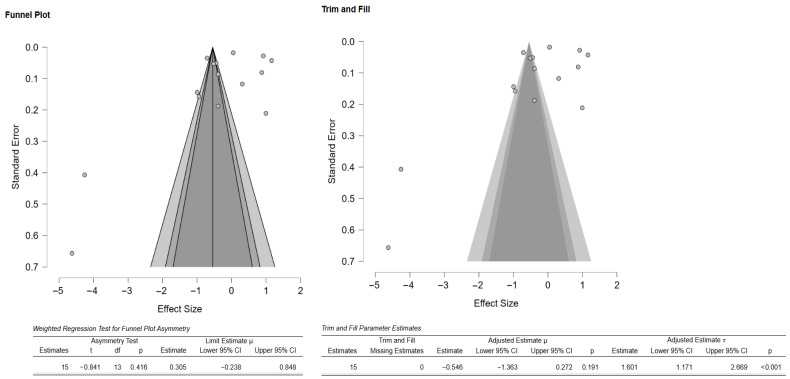
Funnel plot depicting the standard error by mean of the composite outcome. The black dots in the trim-and-fill analysis indicate the studies identified in the systematic review, and the white dots represent the new studies that were imputed using the Duval and Tweedie trim-and-fill method. CI: confidence interval.

As there were few studies for endpoint-specific analysis, the single-study-level composite outcome was calculated. The analysis revealed no significant difference in the composite mechanical outcome between the 3D-printed groups and the CAD/CAM-milled groups (SMD = −0.55; 95% CI: [−1.47, 0.38]; *p* = 0.23; Figure 10). However, there was very high heterogeneity among the studies, which is in line with the results pertaining to the individual outcomes. Visual inspection of the funnel plot suggested some asymmetry; however, Egger’s test did not reveal any statistically significant bias, and the trim-and-fill analysis imputed no studies and showed minimal impact on the pooled estimate (Figure 11). Thus, there is no consistent evidence that 3D-printed zirconia differs from milled zirconia in overall mechanical performance, although results are heterogeneous and likely moderated by printing technology, specimen design, surface finishing, and other factors. However, it should be noted that interpretation of bias results is limited by the very high heterogeneity and the composite nature of the endpoint (different mechanical properties with different scales pooled via SMD), both of which can induce funnel asymmetry and reduce the power and specificity of Egger’s and trim-and-fill analyses.

## 4. Discussion

The present systematic review and meta-analysis compared various mechanical properties (flexural strength, microhardness, density, etc.) and bond strength between CAD/CAM-milled and 3D-printed dental zirconia restorations to provide comprehensive insights into the current state of knowledge in this field. The results of the review and meta-analysis suggest that while CAD/CAM-milled zirconia restorations are reliable and have consistent mechanical properties, 3D-printed ones also have comparable mechanical properties and bond strength. However, there are certain limitations with respect to the manufacturing processes and material properties. For example, the mechanical properties, especially flexural strength and fracture toughness, tends to be slightly better for CAD/CAM-milled zirconia than for 3D-printed zirconia. This observation aligns with the findings of previous studies suggesting that CAD/CAM-milled zirconia is more reliable for dental restorations [19,27]. That said, the results of my review and meta-analysis also suggest that 3D-printed zirconia restorations have reached the clinically acceptable range: some studies showed that in certain conditions or orientations, printed zirconia had comparable or even superior mechanical properties compared to milled restorations. This is especially notable because of the inherent challenges surrounding 3D printing: porosity, microcracks, and layer bonding issues, which can adversely affect the mechanical properties.

The variability noted in the mechanical parameters of 3D-printed zirconia highlights the importance of fine-tuning the manufacturing processes and parameters to minimize the presence of defects and improve performance consistency. Previous studies that assessed bonding and adhesion parameters noted that 3D-printed zirconia could achieve resin cement adhesion that was comparable to that of milled zirconia samples; these studies also noted that surface treatments (e.g., TSC) positively influenced the restorations’ bond strength [7,17]. These findings are important, as they indicate that 3D-printed zirconia restorations with appropriate surface conditioning may be a good option for cases requiring strong adhesive bonding.

Furthermore, the literature suggests that AM is associated with greater porcelain bond strength than SM; thus, 3D-printed zirconia restorations can be used in cases where esthetic considerations are more important than functional ones. Additionally, regarding long-term durability and esthetic, both milled and printed zirconia restorations show clinically acceptable properties after aging, but printed zirconia appears to be more susceptible to degradation, particularly in terms of fracture toughness and bonding reliability [15]. Further, the effect of printing orientation on the stability of mechanical parameters and bonding strength following aging underscores the need for manufacturers to choose the appropriate manufacturing technique and parameters for the production of 3D-printed zirconia restorations. The present meta-analysis also strengthens the conclusion that there is no significant difference in Vickers microhardness between CAD/CAM-milled and 3D-printed zirconia restorations, but it should be noted that there was high heterogeneity among the included studies. This suggests that the differences in the mechanical parameters as well as bonding strength between the two manufacturing methods may not be as pronounced as previously thought, when optimal manufacturing conditions and parameters are used.

Regarding trueness and accuracy, both techniques appear to be associated with acceptable trueness and precision [28]. That said, interestingly, 3D-printed crowns showed better trueness on occlusal and axial surfaces, whereas milled crown samples had better trueness on fitting surfaces [28]. Additionally, in the comparative study by Giugliano et al., milled zirconia disks exhibited more precise dimensions (diameter and thickness) and, thus, were involved in more accurate restorations [19]. Moreover, both techniques resulted in restorations that had clinically acceptable margins in a previous study; the marginal gap was overall smaller in milled samples than in printed ones [29]. Two studies also found that the internal fit was comparable for these two techniques and that they both met the clinical requirements in terms of internal fit [9,30]. Thus, the choice between these two techniques can depend on the clinical requirements: for example, 3D-printed zirconia has better precision when considering complex shapes or narrow areas, whereas milled restorations appear to have better dimensional consistency and translucency [31].

There are advances in the 3D printing technology that could improve the mechanical parameters of zirconia restorations. The literature on this topic has shown that print orientation during manufacturing can influence the mechanical properties of zirconia. Miura et al. showed that a perpendicular orientation was associated with a higher flexural strength than other orientations [32]. Thus, it is evident that printing orientation plays a significant role in enhancing durability. In addition, the use of the DLP technology can help produce high-strength, semi-translucent zirconia ceramics. Thus, DLP, by using UV-curable formulations with optimal zirconia content, can produce fully dense, crack-free ceramics [33]. Additionally, it has been shown that adding silane coupling agents to zirconia suspensions can improve the hardness, material density, and biaxial flexural strength; it also leads to a denser microstructure and, thereby, improve the overall mechanical integrity of 3D-printed zirconia [34]. Further, optimizing various parameters—layer height, print speed, and extrusion temperature—can considerably influence the mechanical parameters of printed zirconia materials like tensile strength and flexural modulus [35]. It has also been reported that using new ceramic formulations and combinations of filler—different zirconia-tetragonal forms and their improvements in thermal treatments and compositions—can improve printability and sintered density zirconia [34]. I believe that these innovations in the 3D printing technology and material science as well as similar advances in the near future can significantly improve the mechanical parameters of zirconia restorations and widen its application in dentistry and other industries requiring high strength and precision.

Clinical variability—including saliva composition and flow, biofilm ecology, occlusal loading patterns/parafunction, antagonist material, soft-tissue phenotype, and host inflammatory milieu—can influence wear, veneer chipping, and peri-restorative tissue response [36]. The in vitro comparisons included in this meta-analysis isolate manufacturing effects; however, it has to be mentioned that they cannot fully account for patient-level heterogeneity. Thus, these factors highlight cautious translation: milled zirconia’s higher predictability (Weibull modulus) may confer clinical reliability [25], whereas printed zirconia’s performance will likely depend on the optimization of build orientation, densification, and finishing to offset variability [37]. Furthermore, evidence on full-contour restorations further shows that substrate stiffness and wall thickness strongly influence fracture resistance, again highlighting the importance of context-dependent clinical performance rather than a single material effect [26].

It should be noted that this study had certain limitations. The high heterogeneity observed among the studies in the meta-analysis of some parameters was a major limitation. Although moderation analyses were attempted, none of the prespecified moderators (e.g., specimen shape, indentation load, testing method, and AM technique) could explain the heterogeneity. It is possible that factors beyond these prespecified ones (e.g., build orientation, paste systems used in the AM procedure, yttria content, grain size, porosity, and sintering/aging protocols) caused the heterogeneity; however, these factors could not be evaluated because they were not reported in some studies. Additionally, for some parameters such as bond strength and fracture resistance, only two studies could be included in the meta-analysis; this limited the statistical power and the ability to draw definitive conclusions. Additionally, it was difficult to assess the associations between mechanical outcomes and bonding strength for dental zirconia in terms of biocompatibility based on surface roughness and porosity because only two studies report the surface characteristics and bonding strength outcomes adequately. Once many studies do a comparative analysis of these parameters in the future, future meta-analyses should aim to include larger numbers of studies in all comparison categories to make the findings robust.

## 5. Conclusions

While the systematic review and meta-analysis findings, within the limitations of the study, reveal that CAD/CAM-milled zirconia often exhibits slightly superior mechanical properties such as strength, hardness, and accuracy, it also highlights that advancements in the 3D-printing technology have begun to reduce this gap. Nevertheless, 3D-printed zirconia has significant potential for clinical applications, particularly where complex shapes are required or lower material wastage is desired. I believe that further research and technological improvements will likely enhance the application of 3D-printed zirconia in the dental field.

## Figures and Tables

**Table 1:** Specific data (values of various parameters) of the selected articles.

Study	Density (g/cm^3^)		Microhardness (GPa)		Flexural Strength (MPa)		Fracture Toughness (MPa·m^1/2^)		Fracture Resistance (N)		Bond Strength (MPa)		Surface Roughness (Sa, µm)		Weibull Modulus *m*		Weibull Characteristic Strength *σ*_0_ (MPa)	
	CAD/CAM	3D Print	CAD/CAM	3D Print	CAD/CAM	3D Print	CAD/CAM	3D Print	CAD/CAM	3D Print	CAD/CAM	3D Print	CAD/CAM	3D Print	CAD/CAM	3D Print	CAD/CAM	3D Print
Abualsaud et al. (2022) [13]	6.065 ± 0.116	5.969 ± 0.201	15.18 ± 0.61	16.08 ± 0.81	1507.27 ± 340.1	839.72 ± 333.13												
Alageel et al. (2025) [14]	5.81 ± 0.17	5.84 ± 0.11	12.15 ± 0.31	16.62 ± 0.42									0.170 ± 0.017	0.159 ± 0.002				
Alhotan et al. (2024) [15]			13.91 ± 0.25	14.06 ± 0.32			4.48 ± 0.112	5.55 ± 1.19										
Bergler et al. (2022) [16]					936.3 ± 255.0	855.4 ± 112.6												
Bömicke et al. (2024) [17]											27.54 ± 10.58	28 ± 8.53						
Cho et al. (2025) [18]			14.45 ± 0.6	14.61 ± 0.49														
Giugliano et al. [19]	6.08 ± 0.01	6.06 ± 0.02			996.16 ± 137.37	845.75 ± 266.16									7.27	7.23/1.92		
Hajjaj et al. (2024) [7]			14.89 ± 1.87	13.13 ± 0.48	1202 ± 174.7	247.7 ± 46.6												
Hassan et al. (2025) [20]			16.25 ± 0.32	13.29 ± 0.33					2786.3 ± 160.5	2693.6 ± 225.6					19.5	12.1	2859.1	2798.6
Hetzler et al. (2025) [21]			15.17 ± 0.27	15.59 ± 0.24	848.6 ± 128.5	879.5 ± 182.2												
Kim et al. (2022) [22]			11.6 ± 0.6	11.5 ± 0.4	927.4 ± 134.1	865.7 ± 148.5	4.9 ± 0.3	4.7 ± 0.4							7.7 (95% CI: 6.9–8.5)	6.4 (95% CI: 5.8–7.0)	984.6	928.5
Moon et al. (2022) [23]											30.26 ± 5.2	35.12 ± 4.09						
Refaie et al. (2023) [24]									1790.8 ± 206	1484.4 ± 369.5								
Zenthöfer et al. (2022) [25]					1461.5 ± 113	1296.5 ± 306.7									15.14 ± 0.84	4.58 ± 0.86	1512.5 ± 2.12	1424.5 ± 80.10
Zenthöfer et al. (2024) [26]									1495.17 ± 256	1801.11 ± 265.22								

CAD/CAM: computed-aided design/computed-aided manufacturing, CI: confidence interval. Note: The combined averages of subgroups have been presented for some studies in this table.

**Table 2:** Findings of the risk-of-bias assessment conducted using the RoBDEMAT tool.

Study	D1.1	D1.2	D1.3	D2.1	D2.2	D3.1	D3.2	D4.1	D4.2
Abualsaud et al. (2022) [13]	A	N	A	A	A	A	N	A	A
Alhotan et al. (2024) [15]	A	N	A	A	A	A	N	A	A
Alageel et al. (2025) [14]	A	N	A	A	A	A	N	A	A
Bergler et al. (2020) [16]	A	N	A	I	A	A	N	A	A
Bömicke et al. (2024) [17]	A	N	N	I	A	A	N	A	A
Cho et al. (2025) [18]	A	N	A	A	A	A	N	A	A
Giugliano et al. (2023) [19]	A	N	A	A	A	A	A	A	A
Hajjaj et al. (2024) [7]	A	N	A	A	A	A	N	A	A
Hassan et al. (2025) [20]	A	N	A	A	A	A	N	A	A
Hetzler et al. (2025) [21]	A	N	A	A	A	A	N	A	A
Kim et al. (2022) [22]	A	N	N	A	A	A	N	A	A
Moon et al. (2022) [23]	A	N	N	I	A	A	N	A	A
Refaie et al. (2023) [24]	A	N	A	I	A	A	N	A	A
Zenthöfer et al. (2022) [25]	A	A	A	A	A	A	N	A	A
Zenthöfer et al. (2024) [26]	A	N	A	A	A	A	N	A	A

Domains of Bias: D1—Bias in planning and allocation: D1.1 Control group; D1.2 Sample randomization; D1.3 Sample size rationale and reporting. D2—Bias in sample/specimen preparation: D2.1 Standardization of samples and materials; D2.2 Identical experimental conditions across groups. D3—Bias in outcome assessment: D3.1 Adequate and standardized testing procedures and outcomes; D3.2 Blinding of the test operator. D4—Bias in data treatment and outcome reporting: D4.1 Statistical analysis; D4.2 Reporting study outcomes [11]. Rating Scale: A = Adequately performed/reported; I = Insufficiently performed/reported; N = Not performed/reported. RoBDEMAT: Risk of Bias tool for Pre-Clinical Dental Material Research

**Table 3:** Findings of the risk-of-bias assessment conducted using the modified CONSORT tool.

Study	1	2a	2b	3	4	5	6	7	8	9	10	11	12	13	14
Abualsaud et al. (2022) [13]	*	*	*	*	*	*					*	*	*	*	
Alhotan et al. (2024) [15]	*	*	*	*	*	*					*	*	*	*	
Alageel et al. (2025) [14]	*	*	*	*	*	*					*	*	*	*	
Bergler et al. (2020) [16]	*	*	*		*	*					*	*	*	*	
Bömicke et al. (2024) [17]	*	*	*		*						*	*	*	*	
Cho et al. (2025) [18]	*	*	*	*	*	*					*	*	*	*	
Giugliano et al. (2023) [19]	*	*	*	*	*	*				*	*	*	*	*	
Hajjaj et al. (2024) [7]	*	*	*	*	*	*					*	*	*	*	
Hassan et al. (2025) [20]	*	*	*	*	*	*					*	*	*	*	
Hetzler et al. (2025) [21]	*	*	*	*	*	*					*	*	*	*	
Kim et al. (2022) [22]	*	*	*	*	*						*	*	*	*	
Moon et al. (2022) [23]	*	*	*		*						*	*	*	*	
Refaie et al. (2023) [24]	*	*	*		*	*					*	*	*	*	
Zenthöfer et al. (2022) [25]	*	*	*	*	*	*	*	*			*	*	*	*	
Zenthöfer et al. (2024) [26]	*	*	*	*	*	*					*	*	*	*	

Modified CONSORT Checklist: Abstract: Item 1—Structured summary of trial design, methods, results, and conclusions. Introduction: Item 2a—Scientific background and explanation of rationale; Item 2b—Specific objectives and/or hypotheses. Methods: Item 3—Intervention for each group, with sufficient detail for replication; Item 4—Defined, pre-specified primary and secondary outcome measures, including how and when they were assessed; Item 5—How sample size was determined; Item 6—Method used to generate the random allocation sequence; Item 7—Mechanism used to implement the random allocation sequence, and steps to conceal allocation until intervention assignment; Item 8—Who generated the allocation sequence, who enrolled specimens, and who assigned interventions; Item 9—If blinding was performed, who was blinded after assignment to intervention and how; Item 10—Statistical methods used to compare groups for primary and secondary outcomes. Results: Item 11—Results for each primary and secondary outcome, for each group, with effect size and precision. Discussion: Item 12—Trial limitations, addressing sources of potential bias, imprecision, and generalizability. Other Information: Item 13—Sources of funding and other support, and role of funders; Item 14—Where the full trial protocol can be accessed, if available [12]. * denotes that the item was reported adequately in the study.

## Data Availability

No new data were created or analyzed in this study. Data sharing is not applicable to this article.

## Supplementary Materials

The following supporting information can be downloaded at: https://www.mdpi.com/article/10.3390/ma18225112/s1 [38].

## Abbreviations

The following abbreviations are used in this manuscript:

CAD/CAM	Computer-Aided Design/Computer-Aided Manufacturing
3D	Three-Dimensional
SM	Subtractive Manufacturing
SLA	Stereolithography
SLS	Selective Laser Sintering
FDM	Fused Deposition Modeling
DLP	Digital Light Processing
SLM	Selective Laser Melting
DIP	Direct Inkjet Printing
YSZ	Yttria-Stabilized Zirconia
Y-TZP	Yttria-stabilized Tetragonal Zirconia Polycrystals
3Y-TZP/5Y-PSZ	3 mol% Yttria Tetragonal Zirconia Polycrystals/5 mol% Yttria Partially Stabilized Zirconia
PRISMA	Preferred Reporting Items for Systematic Reviews and Meta-Analyses
PICO	Population, Intervention, Comparison, Outcomes
RoBDEMAT	Risk of Bias tool for Pre-Clinical Dental Material Research
CONSORT	Consolidated Standards of Reporting Trials
JASP	Jeffreys’s Amazing Statistics Program (statistical software)
SEM	Scanning Electron Microscope
ISO	International Organization for Standardization
SMD	Standardized Mean Difference
CI	Confidence Interval
AM	Additive Manufacturing
HV	Vickers Hardness
MPa	Megapascal (unit of pressure/stress)
MPa·m½	Megapascal square root meter (unit of fracture toughness)
TSC	Tribochemical Silica Coating
